# Supplementary material for: Time-series transcriptome analysis identified differentially expressed genes in broiler chicken infected with mixed Eimeria species
Source: Front Genet. 2022 Aug 8;13:886781. doi: 10.3389/fgene.2022.886781 (PMC9393255; doi:10.3389/fgene.2022.886781)
Supplement: Supplementary file 2 [file DataSheet1.ZIP › 7dpi_GO.Gsea.1625106495923/GOBP_OXIDATIVE_PHOSPHORYLATION.html]

Details for gene set GOBP\_OXIDATIVE\_PHOSPHORYLATION[GSEA]

|  || Dataset | TMM\_7dpi\_gct\_format\_7dpi\_gct\_format.Class\_4dpi.cls #PC\_versus\_NC.Class\_4dpi.cls #PC\_versus\_NC\_repos |
| Phenotype | Class\_4dpi.cls#PC\_versus\_NC\_repos |
| Upregulated in class | 1 |
| GeneSet | GOBP\_OXIDATIVE\_PHOSPHORYLATION |
| Enrichment Score (ES) | 0.72651285 |
| Normalized Enrichment Score (NES) | 3.5598385 |
| Nominal p-value | 0.0 |
| FDR q-value | 0.0 |
| FWER p-Value | 0.0 |
Table: GSEA Results Summary

  

Fig 1: Enrichment plot: GOBP\_OXIDATIVE\_PHOSPHORYLATION      
 Profile of the Running ES Score & Positions of GeneSet Members on the Rank Ordered List

  

| SYMBOL | TITLE | RANK IN GENE LIST | RANK METRIC SCORE | RUNNING ES | CORE ENRICHMENT || 1 | DGUOK | na | 238 | 1.001 | 0.0051 | Yes |
| 2 | COX5A | na | 300 | 0.942 | 0.0236 | Yes |
| 3 | NDUFA2 | na | 303 | 0.935 | 0.0468 | Yes |
| 4 | NDUFB6 | na | 307 | 0.933 | 0.0699 | Yes |
| 5 | NDUFS5 | na | 308 | 0.932 | 0.0933 | Yes |
| 6 | CHCHD10 | na | 312 | 0.929 | 0.1163 | Yes |
| 7 | NDUFB7 | na | 323 | 0.915 | 0.1384 | Yes |
| 8 | NDUFB2 | na | 348 | 0.885 | 0.1585 | Yes |
| 9 | NDUFAB1 | na | 356 | 0.876 | 0.1799 | Yes |
| 10 | NDUFA7 | na | 371 | 0.857 | 0.2002 | Yes |
| 11 | NDUFC2 | na | 394 | 0.834 | 0.2192 | Yes |
| 12 | UQCR10 | na | 444 | 0.785 | 0.2348 | Yes |
| 13 | CYCS | na | 475 | 0.763 | 0.2513 | Yes |
| 14 | COX6A1 | na | 480 | 0.760 | 0.2700 | Yes |
| 15 | UQCR11 | na | 485 | 0.754 | 0.2886 | Yes |
| 16 | UQCRB | na | 487 | 0.753 | 0.3074 | Yes |
| 17 | NDUFA11 | na | 497 | 0.746 | 0.3253 | Yes |
| 18 | COX7A2 | na | 499 | 0.743 | 0.3438 | Yes |
| 19 | NDUFA13 | na | 557 | 0.708 | 0.3568 | Yes |
| 20 | NDUFS3 | na | 623 | 0.675 | 0.3682 | Yes |
| 21 | NDUFB3 | na | 656 | 0.657 | 0.3820 | Yes |
| 22 | NDUFS8 | na | 661 | 0.653 | 0.3980 | Yes |
| 23 | NDUFS6 | na | 686 | 0.642 | 0.4121 | Yes |
| 24 | SDHD | na | 708 | 0.632 | 0.4261 | Yes |
| 25 | UQCC3 | na | 735 | 0.620 | 0.4395 | Yes |
| 26 | NDUFA1 | na | 742 | 0.616 | 0.4544 | Yes |
| 27 | UQCRHL | na | 788 | 0.594 | 0.4655 | Yes |
| 28 | NDUFA5 | na | 794 | 0.593 | 0.4799 | Yes |
| 29 | GHITM | na | 842 | 0.575 | 0.4904 | Yes |
| 30 | DNAJC15 | na | 863 | 0.566 | 0.5029 | Yes |
| 31 | NDUFB5 | na | 902 | 0.554 | 0.5136 | Yes |
| 32 | SDHAF2 | na | 919 | 0.550 | 0.5260 | Yes |
| 33 | COX6C | na | 930 | 0.546 | 0.5388 | Yes |
| 34 | COQ9 | na | 939 | 0.541 | 0.5517 | Yes |
| 35 | NDUFV3 | na | 988 | 0.527 | 0.5609 | Yes |
| 36 | NDUFB4 | na | 1015 | 0.517 | 0.5717 | Yes |
| 37 | NDUFA12 | na | 1023 | 0.513 | 0.5839 | Yes |
| 38 | COX7B | na | 1055 | 0.501 | 0.5939 | Yes |
| 39 | PPIF | na | 1060 | 0.500 | 0.6061 | Yes |
| 40 | NDUFA6 | na | 1079 | 0.492 | 0.6169 | Yes |
| 41 | NDUFA8 | na | 1080 | 0.492 | 0.6292 | Yes |
| 42 | UQCRQ | na | 1103 | 0.484 | 0.6395 | Yes |
| 43 | NDUFS2 | na | 1124 | 0.477 | 0.6497 | Yes |
| 44 | COX4I1 | na | 1125 | 0.476 | 0.6617 | Yes |
| 45 | SDHC | na | 1135 | 0.473 | 0.6728 | Yes |
| 46 | NDUFB10 | na | 1215 | 0.450 | 0.6774 | Yes |
| 47 | UQCRFS1 | na | 1263 | 0.437 | 0.6844 | Yes |
| 48 | ATP5PB | na | 1267 | 0.436 | 0.6951 | Yes |
| 49 | NDUFV1 | na | 1307 | 0.428 | 0.7025 | Yes |
| 50 | NDUFB8 | na | 1330 | 0.423 | 0.7113 | Yes |
| 51 | NDUFS7 | na | 1371 | 0.412 | 0.7182 | Yes |
| 52 | NDUFV2 | na | 1395 | 0.408 | 0.7265 | Yes |
| 53 | BID | na | 1694 | 0.334 | 0.7099 | No |
| 54 | UQCRC2 | na | 1700 | 0.332 | 0.7178 | No |
| 55 | COX7A2L | na | 1761 | 0.316 | 0.7207 | No |
| 56 | NDUFB9 | na | 1842 | 0.299 | 0.7214 | No |
| 57 | COX15 | na | 1927 | 0.282 | 0.7215 | No |
| 58 | NDUFB1 | na | 1995 | 0.272 | 0.7227 | No |
| 59 | TAZ | na | 2298 | 0.223 | 0.7029 | No |
| 60 | UQCRC1 | na | 2326 | 0.217 | 0.7061 | No |
| 61 | NDUFA4 | na | 2726 | 0.160 | 0.6767 | No |
| 62 | NDUFA9 | na | 2879 | 0.141 | 0.6674 | No |
| 63 | ISCU | na | 2900 | 0.138 | 0.6692 | No |
| 64 | PDE12 | na | 2905 | 0.138 | 0.6723 | No |
| 65 | COX10 | na | 3408 | 0.073 | 0.6321 | No |
| 66 | NDUFA10 | na | 3412 | 0.073 | 0.6337 | No |
| 67 | SDHA | na | 3488 | 0.064 | 0.6290 | No |
| 68 | NDUFS1 | na | 3763 | 0.033 | 0.6068 | No |
| 69 | PARK7 | na | 3768 | 0.032 | 0.6073 | No |
| 70 | NDUFAF1 | na | 3921 | 0.014 | 0.5949 | No |
| 71 | SURF1 | na | 3926 | 0.013 | 0.5949 | No |
| 72 | CDK1 | na | 4341 | -0.035 | 0.5610 | No |
| 73 | NDUFS4 | na | 4402 | -0.040 | 0.5570 | No |
| 74 | AK4 | na | 4515 | -0.052 | 0.5489 | No |
| 75 | COX7C | na | 4881 | -0.090 | 0.5206 | No |
| 76 | SNCA | na | 5117 | -0.114 | 0.5037 | No |
| 77 | DLD | na | 5596 | -0.158 | 0.4676 | No |
| 78 | STOML2 | na | 5602 | -0.159 | 0.4712 | No |
| 79 | NIPSNAP2 | na | 5689 | -0.168 | 0.4681 | No |
| 80 | SLC25A33 | na | 5797 | -0.179 | 0.4637 | No |
| 81 | MSH2 | na | 7352 | -0.347 | 0.3420 | No |
| 82 | FXN | na | 7832 | -0.399 | 0.3119 | No |
| 83 | MLXIPL | na | 8034 | -0.426 | 0.3057 | No |
| 84 | CCNB1 | na | 8681 | -0.511 | 0.2643 | No |
| 85 | VCP | na | 9238 | -0.589 | 0.2324 | No |
Table: GSEA details [plain text format]

  

Fig 2: GOBP\_OXIDATIVE\_PHOSPHORYLATION      
 Blue-Pink O' Gram in the Space of the Analyzed GeneSet

  

Fig 3: GOBP\_OXIDATIVE\_PHOSPHORYLATION: Random ES distribution      
 Gene set null distribution of ES for **GOBP\_OXIDATIVE\_PHOSPHORYLATION**
